# Atopic dermatitis: a comprehensive updated review of this intriguing disease with futuristic insights

**DOI:** 10.1007/s10787-025-01642-z

**Published:** 2025-02-07

**Authors:** Heidi M. Abdel-Mageed

**Affiliations:** Molecular Biology Department, National Research Centre, El Behoth St, Dokki, Giza, Egypt

**Keywords:** Atopic dermatitis, Artificial intelligence, Skin, Nanodelivery, Immune dysfunction, Biological therapy, JAK inhibitors, ChatGPT

## Abstract

Atopic dermatitis (AD) is a paradigmatic prevalent, long-lasting, and inflammatory skin condition with a diverse range of clinical manifestations. The etiology and clinical symptoms of AD are influenced by complex pathophysiological processes, which involve a strong genetic component, epidermal dysfunction, and immunological dysregulation, and a strong influence of other physiological and environmental factors. The FDA has approved targeted and well-tolerated immunomodulators including biologics like dupilumab and crisaborole, and small molecules such as baricitinib, as novel therapies for AD. They effectively treat AD but are too expensive for most patients. The review provides an update on the state of knowledge of AD pathogenesis, discusses the available diagnostic and scoring indices, and provides a scientific foundation for treatment methods for AD. This review also presents data on clinical efficacy of innovative treatments’ considering recent guidelines, emphasizing the newest medications and ongoing trials. Finally, the new implication of artificial intelligence (AI) in AD management is explored, where AI can speed up diagnosis and therapy. The PubMed, Google Scholar, and ScienceDirect databases were used for this review.

**Graphical Abstract:**

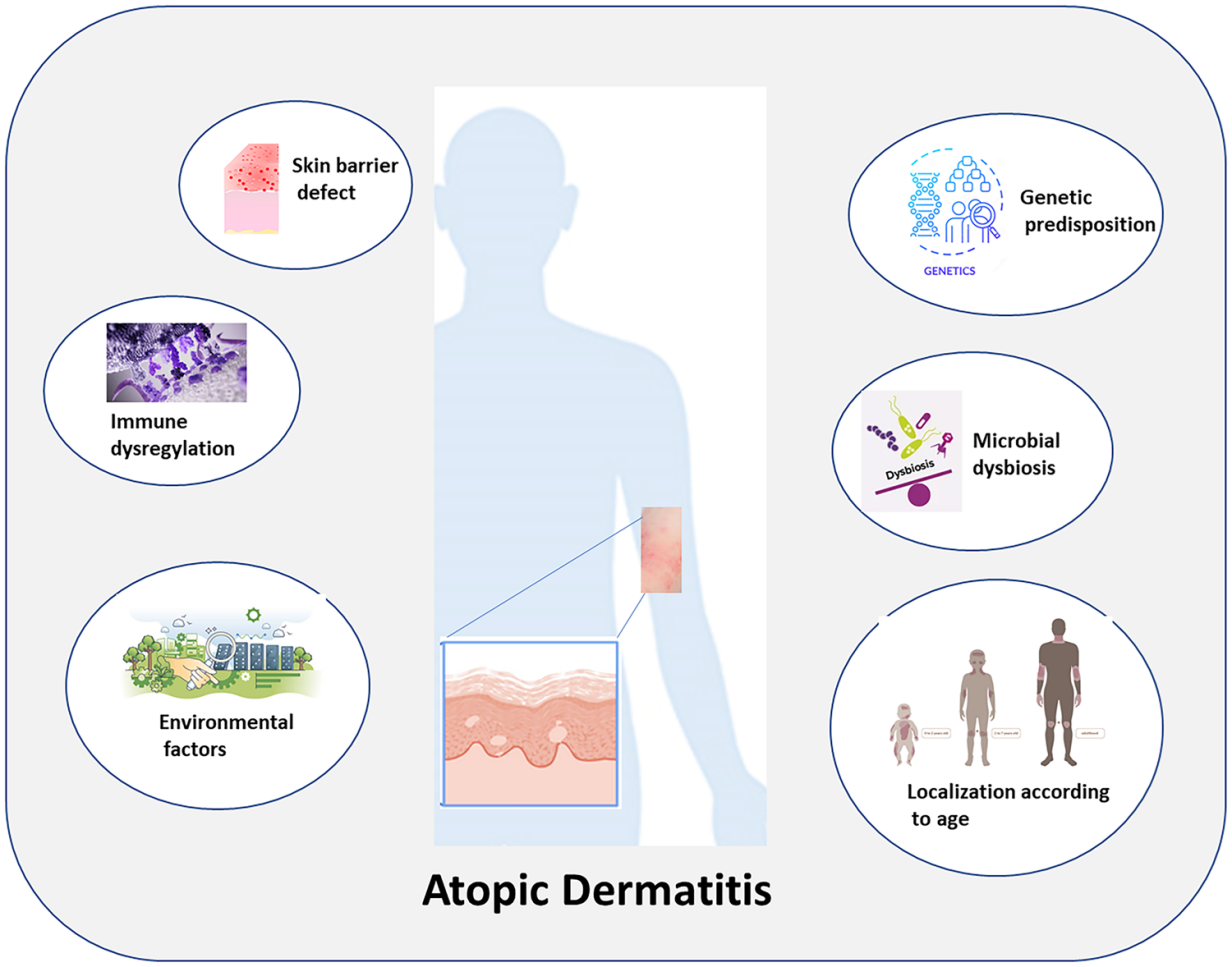

## Introduction

Atopic dermatitis (AD), also known as atopic eczema, is a chronic inflammatory skin disease that can hinders skin function. AD is characterized by pruritus that worsens at night, recurrent eczematous lesions, skin pain, and a fluctuating course. In general, the course of the disease varies greatly and each patient’s pathway is unique. AD is a member of the “atopic triad” with food allergy, allergic rhinitis, and bronchial asthma. AD is ranked 15th in terms of the global disease burden (Afshari et al. 2024). New findings indicate that infantile AD is closely linked to the subsequent onset of childhood asthma, allergic rhinitis, and food allergies, collectively known as the “atopic march” (Silverberg et al. 2021; Afshari et al. 2024).

There is a substantial socioeconomic cost associated with AD, which significantly lowers the quality of life for patients and their families (Barnes 2010). Sleep disturbance, anxiety, hyperactivity, and depression are usually associated with atopic dermatitis (Silverberg et al. 2021). The majority of disease cases (80 %) begin in childhood or infancy, with the remaining cases occurring at maturity. Worldwide prevalence varies between 2% and 10% in adults and between 2.7% and 20% in children. AD prevalence has grew by 0.98% per decade in adolescents and by 1.21% per decade in children globally (Langan et al. 2023). Despite recent advancements, a “one-size-fits-all” approach to disease care remains prevalent, and current therapeutic options are limited. AD management is complicated by long-term adverse effects and individual responses. AD patients experience additional challenges due to high pharmaceutical costs and limited specialty care access. (Bieber et al. 2021).

This review addresses the different parameters underlying AD pathogenesis to, gain a deeper understanding of the immune response sequence and epidermal barrier dysfunction mechanisms that underpin chronic inflammatory reaction. Additionally, environmental and physiological parameters of AD pathogenesis are explored. Next, diagnostic approaches and scoring indices are identified. In addition, this review discusses recent drug approvals and ongoing trials. When consistently administered, new biologics such as JAK inhibitors (JAKi) continue to prove effective and beneficial over the long term. In addition to traditional medical approaches, this review covers nonmedical approaches. This review concludes by examining how artificial intelligence (AI) can optimize AD management through database skills to enhance referral efficiency and diagnosis accuracy.

## Atopic dermatitis pathophysiology

The exact etiopathogenesis of AD remains unknown. Immunological, environmental, and genetic variables are major contributors to its development. According to a recent meta-analysis, children with at least one parent with a history of AD were approximately 40% more likely to develop AD. When both parents have atopic disorders, the likelihood increases to 60%–80% (Ravn et al. 2020). Additionally, although no particular gene locus was found, genome-wide linkage studies have discovered possible correlations with AD on several chromosomes, including chromosomes 1, 3, 5, 11, 15, and 17 (Brunner et al. 2017).

### Disease biomarkers and abnormal immunological responses

**Figure 1:**
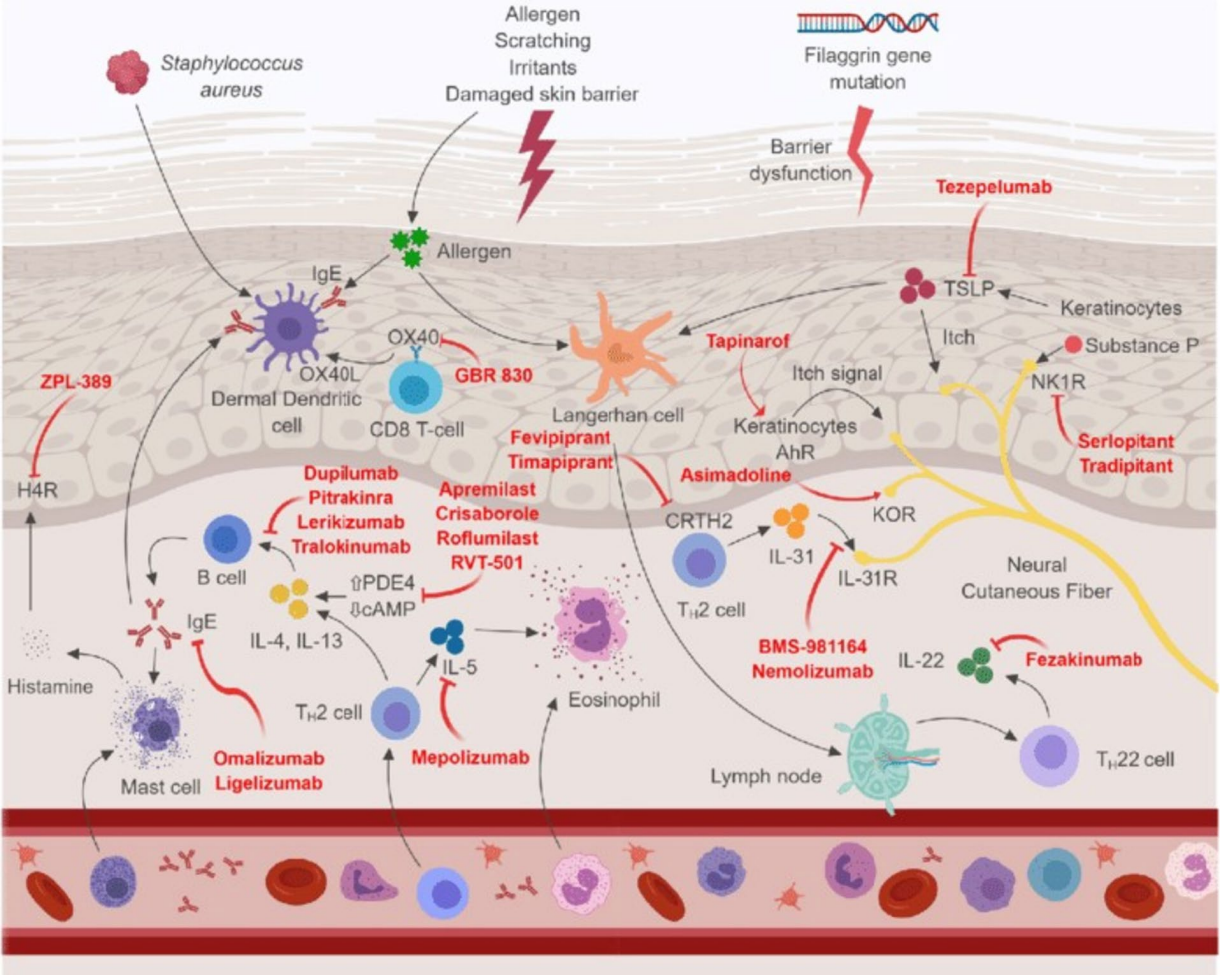
An outline of atopic dermatitis pathogenesis and mechanism of action of therapeutic agents. Reproduced with permission from Nguyen et al. (2019). CRTH2 chemoattractant receptor homologs molecule expressed on TH2 cells, AhR aryl hydrocarbon receptor, IgE immunoglobulin-E, IL interleukin, and H4R histamine-4 receptor JAK/STAT PDE4 phosphodiesterase 4, R receptor, KOR κ-opioid receptor, NK1R neurokinin-1 receptor, Janus kinase/signal transducer and activator of transcription, and TSLP thymic stromal lymphopoietin

Progress in AD pathophysiology has revealed that systemic inflammation and immunological dysfunction are significant disease mediators (Fig. 1). Acute versus chronic eczematous skin lesions typically show clinically and distinctively in terms of dermal color (bright versus dull red), thickness (flatter versus thicker), and spongiosis (marked versus minor). AD is distinguished by strong production of type 2 cytokines, such as interleukin (IL)-4, IL-5, IL-13, and IL-31, and abnormal and excess Th2 cell count and ILC2 activation. All patients with AD exhibit a type 2 and type 22 immune responses, but cytokine activation varies, including that of IL-22, IL-17, IL-9, and IFN-γ, contributing to various clinical AD subtypes (Brunner et al. 2017). Likewise, studies on Childhood AD demonstrated increased type 2 inflammation, but higher type 9 and type 17 effector activation at disease onset (Esaki et al. 2016).

The pathophysiology of AD is affected by IL-4 and IL-13, which are important mediators of the type 2 pathway. These effects include the spread of inflammation, epidermal barrier disruption, and itching. The itch-promoting cytokine IL-31 has been linked to illness severity, and serum levels of IL-13 are positively correlated with both cytokine levels. Intriguingly, IL-22 and IL-17, a type 17 cytokine (IL-17), upregulate the production of several S100 epidermal development proteins. These include S100A7, S100A8, and S100A9 (Moyle et al. 2019). These S100 proteins were significantly upregulated in acute lesions compared with non-lesional skin, and were further upregulated in chronic lesions, along with IL-31 and IL-22. Increased expression of related cytokines (e.g., IL-13 and IL-31 [type 2], IL-22 [type 22]) indicates that type 2- and type 22-specific inflammation is often enhanced in chronic lesions compared with acute lesions (Gittler et al. 2012; Moyle et al. 2019). There is conflicting information on IL-4 gene expression changes in chronic lesions; some findings indicate a decrease in expression (Gittler et al. 2012), while others claim an increase (Silverberg and Kantor 2017).

### Defective skin barrier and dysregulated immune responses

The skin barrier anxiously guards the body from external hazards such as, pathogens, chemicals, irritants, and allergens, from, entering the inner layers. According to the “Inside-out” concept, immune responses to irritants and allergens degrade the skin barrier in AD patients (Dähnhardt‐Pfeiffer et al. 2013). Table 1 presents a comparison of normal and atopic dermatitis (AD)-affected skin that emphasizes the fundamental distinctions between the two, highlighting the complex nature of AD pathogenesis. AD is linked to transepidermal water loss (TEWS), which indicates skin barrier abnormalities. The skin barrier function’s can be measured by calculating the percentage of transepidermal water loss (%TEWL), which is inversely proportional to the diffusional permeation path length. Methods such as stratum corneum cohesion, pH testing, and tracer compound permeability offer valuable information about the integrity and efficacy of the barrier.

**Table 1:** A comparison between normal skin and atopic dermatitis (AD) skin, focusing on various aspects that highlights the key differences between normal and atopic dermatitis-affected skin, emphasizing the multifaceted nature of AD pathophysiology

Aspect	Normal skin	Atopic dermatitis (AD) skin
Skin microbiome	- Diversity: high microbial diversity, which helps maintain skin health. - Dominant species: coagulase-negative staphylococci (CoNS) - *S. aureus* colonization: low levels of *Staphylococcus aureus*. - Microbial balance: a balanced microbiome supports skin barrier function and immune responses.	- Diversity: reduced microbial diversity. - Dominant species: increased colonization by *Staphylococcus aureus*. - S. aureus colonization: high levels of *S. aureus*, which can exacerbate inflammation. - Microbial imbalance: dysbiosis contributes to impaired barrier function and heightened immune responses.
Immune system	- Inflammation: absent; the immune system is in a balanced state. - Innate immunity: functions optimally to protect against pathogens.	- Inflammation: present; characterized by a Th2-predominant inflammatory response. - Innate immunity: suppressed, leading to decreased ability to combat infections.
pH	- Level: acidic, typically around pH 4–5. - Function: supports a healthy microbiome and barrier function.	- Level: elevated to neutral or alkaline levels. - Function: higher pH can disrupt the skin barrier and favor the growth of pathogenic bacteria like *S. aureus*.
Barrier function	- Integrity: intact, effectively preventing water loss and entry of harmful substances. - Water loss: minimal transepidermal water loss (TEWL).	- Integrity: compromised due to genetic factors (e.g., filaggrin mutations) and inflammation. - Water loss: increased TEWL, leading to dry skin. - Microbial entry: enhanced penetration of allergens and microbes, including *S. aureus*, which can further disrupt the barrier. - Mechanical damage: scratching exacerbates barrier dysfunction.

*S. aureus* *Staphylococcus aureus*, *TEWL* transepidermal water loss

The dermis hosts the majority of the skin’s adaptive immune cells, such as memory T lymphocytes. There are two types of T lymphocytes: cytotoxic and T helper. Cytotoxic T lymphocytes identify surface antigens and initiate the cell-killing process. T-helper lymphocytes help mature dendritic cells and support effector cells through cytokine production (Debes and McGettigan 2019). B lymphocytes are implicated in inflammation and immunosuppression in addition to their passive and active role in preserving immunity in the skin through the generation of certain antibodies. After maturation, B lymphocytes become plasma cells, which reside inside the bone marrow and lymph nodes and manufacture certain antibodies. B lymphocytes are important for host defenses because they produce anti-inflammatory IL-10 and pro-inflammatory cytokines. Additionally, they provide T lymphocytes with efficient antigen-presenting cells and serve as a resource for immunoregulatory cytokines (Afshari et al. 2024).

### Protein filaggrin (FLG)

FLG protein plays a major role in the structural integrity of the stratum corneum’s (SC). FLG promotes the development of a cornified cell envelope adjacent to the corneocyte after the buildup of keratin filaments within the corneocyte (De Lusignan et al. 2021). The ability of healthy skin to store water is attributed to filaggrin. The acidity of stratum corneum is maintained by breakdown into hygroscopic amino acids such as pyrrolidone carboxylic acid and urocanic acid. Staphylococcal surface proteins clumping factor B and fibronectin-binding protein, which bind to host proteins cytokeratin and fibronectin, are expressed less often in the acidic environment of healthy skin (Kakkar et al. 2023). Furthermore, the enzyme responsible for lipid synthesis, inflammation, and desquamation must be kept active by maintaining an acidic pH (Smieszek et al. 2020). On a molecular level, the lack of filaggrin, involucrin, claudins, ceramides, cholesterol, and free fatty acids results in skin barrier defects. A loss-of-function mutation in the FLG gene (FLG LoF) is a major genetic risk factor for AD (De Lusignan et al. 2021). AD skin is more susceptible to allergies and infections upon dehydration due to FLG LoF (Kapur et al. 2018). Filaggrin deficiencies increase pH and lower pyrrolidone and urocanic acid carboxylic acids, promoting Staphylococcus aureus growth’ (Smieszek et al. 2020). 25%–30% of European and Asian AD patients with early-onset AD have FLG LoF. Compared with AD patients without FLG LoF, individuals with FLG LoF showed a sevenfold higher prevalence of 4 or more skin infections requiring antibiotics within a year (Kapur et al. 2018).

### Serine protease activity and pH

The activity of the serine proteases kallikreins (KLK) 5 and 7 is influenced by the pH of the stratum corneum. The proteases KLK5 and KLK7 hydrolyze extracellular corneodesmosomal proteins that simultaneously connect the corneocyte. The enhanced activity of these cells indicate reduced corneocyte adhesion and desquamation (Zani et al. 2021). The increased activity of serine protease in AD indicates the skin’s higher pH (Cruz-Silva et al. 2022).

Another enzyme linked to AD pathogenesis is neutrophil elastase (NE), a broad-spectrum serine protease released by neutrophils and macrophages. It is a potent pro-inflammatory and chemotactic factor generator due to its capacity to degrade various extracellular matrix proteins. Neogenesis externa appears to play a role in the inflammatory elements of eczematous disorders that result in desquamation and spongiosis once the matrix is destroyed down and intercellular connections are lost. Specifically, it has been noted that neutrophil elastase activity is not present in the skin of healthy individuals but is significantly increased in patients with allergic contact dermatitis (average 55-fold) and atopic dermatitis (average 35-fold), the most common kind of eczema (Cruz-Silva et al. 2022).

### Lipid-based matrix functionality

The lipid composition of the stratum corneum significantly varies between AD patients and healthy individuals. Ceramides, free fatty acids, and cholesterol comprise the three primary lipid components in the stratum corneum. The lipid matrix forms tightly packed lipid layers, resulting in a highly structured structure (Nørreslet et al. 2019). The intercellular lipid matrix and epidermal barrier are predominantly crossed through this significant route (Nørreslet et al. 2019). In AD, decreased fatty acid expression impedes elongases, resulting in altered skin lipids, interleukin-4 (IL-4) anomalies, and interleukin-13 (IL-13) abnormalities (Makowska et al. 2023).

### Keratinocytes impaired barrier function

Keratinocytes are skin epithelial cells that participate in immune and barrier functions. Keratinocytes produce more thymic stromal lymphopoietin, IL-33, and IL-25 in patients with AD, which activates ILC2cytokine expression in AD lesions to create IL-4, IL-5, and IL-13(Stier and Peebles 2017). IL-4 and IL-13 minimize keratinocyte antimicrobial peptide expression and skin barrier function (Berdyshev et al. 2018), predisposing patients with AD to skin infections. Endothelial cells, macrophages, mast cells, and basophils produce IL-33 alongside keratinocytes. Furthermore, patients with AD, the stratum corneum becomes thinner due to a lack of terminal keratinocyte differentiation (De Lusignan et al. 2021)).

### Dermal dendritic cells (dDCs)

Dermal dendritic cells (dDCs) are located below the epidermal-dermal junction and have scarcer dendrites than Langerhans cells. As a result of this decline, the immune system can engage more freely. Dermal dendritic cells express IL-10 and epithelial cell adhesion molecules. They use cytokines and chemokines to stimulate B cells to create immunoglobulins, such as IgM, for pathogen immunosurveillance. This activity emphasizes their role in dermal innate and adaptive immunity (Guttman-Yassky et al. 2023). For AD patients, dendritic cell abnormalities may increase their risk of infection. Whereas, the production of interferon-α.2 by both myeloid and plasmacytoid dendritic cells is significantly lower. Additionally, the ability of Langerhans cells and inflammatory dendritic epidermal cells to sense S aureus in AD patients is impaired (Iwamoto et al. 2018). Recently AD patients were shown to lack natural killer cells (NKs) due to a possible natural killer (NKs) cell-type 2 inflammation counter-regulatory mechanism; this deficit may promote type 2 inflammation in AD patients (Mack et al. 2020).

### Innate immune cells

Monocytes, granulocytes, and mast cells are innate immune cells in the dermis. Monocytes are precursors of macrophages, which are highly mobile phagocytic cells capable of initiating immune responses (Hashimoto et al. 2013). Proteases and antimicrobial peptide granules are detected in granulocytes of various species, such as neutrophils, basophils, eosinophils, and mast cells. These cells degranulate in an IgE-dependent manner, and upon activation, granulocytes are attracted to the invasion site. Histamine-containing mast cells are tryptase and chymase-positive, and function as effector cells in IgE-mediated hypersensitivity reactions (Lin and Loré 2017). Furthermore, mast cells mediate the maturation, migration, and antigen presentation of Langerhans cells and cutaneous dendritic cells utilizing TNF-α and histamine production. Mast cells produce TGF-β and IL-10, which stimulate the production of T-regulatory lymphocytes and induce immunological tolerance. TGF-β regulates B-lymphocyte activity by increasing IgA class switching. Additionally, TGF-β drives fibrotic processes in AD patients following inflammation-induced damage (Weissler and Frischmeyer-Guerrerio 2019). The final type of dermal innate immune cells is referred to as natural killers (NK). It shares certain similarities with adaptive immunity. They can also cause apoptosis or release intracellular granules when they bind to pathogens or cancerous cells that have been labeled with IgG (Brillantes and Beaulieu 2020).

### Skin-flora dysbiosis

Immune defense and management are supported by normal skin flora beyond the epithelium. The equilibrium of the skin microbiome is affected by several interrelated environmental factors, including pH, temperature, dryness, host genetics, antibiotic use, and hygiene habits. (Nakatsuji and Gallo 2019). A decrease in bacterial diversity and the dominance of one microbe are hallmarks of “dysbiosis,” which is brought on by the dysregulation of these systems. Commensal bacteria influence the human immune system to reduce inflammation, protect against microbial infections, and directly outcompete *S. aureus*. AD patients lack commensal bacteria, which makes *S. aureus* more virulent in lesion skin (Nakatsuji and Gallo 2019). *S. aureus* colonizes 30% to 100% of AD patients, unlike in healthy people. It is one of the most important environmental variables in AD etiology (Fyhrquist et al. 2019). By deviating both innate and adaptive immune responses through various mechanisms, S. aureus exacerbates skin inflammation and allergic reactions (Wang et al. 2021).

The superantigen staphylococcal enterotoxin B released by *S. aureus* from the skin of AD patients activates lymphocytes and macrophages, causing inflammation (Kim et al. 2019). Enterotoxins break down the skin barrier and increase type 2 inflammation. Superantigens reduce cutaneous interferon-γ and tumor necrosis factor-α (TNF-α) production, which are crucial for cellular protection against bacterial and viral infections (Orfali et al. 2019). Superantigen production is significantly higher in methicillin-resistant *S. aureus* than in methicillin-sensitive *S. aureus*. Atypical barrier and keratinocyte loss in AD may be caused by superantigen and the α toxin. Furthermore, by inducing mast cell degranulation, staphylococcal delta toxin can cause AD-associated inflammation (Orfali et al. 2018). Phenol-soluble modulin (PSM)-α from *S. aureus* increases IL-36α and IL-1α production in keratinocytes, leading to IL-17 production and increased neutrophil recruitment (Liu et al. 2017). Additionally, through Toll-like receptor 2 (TLR2), *S. aureus* induces thymic stromal lymphopoietin production and mast cell degranulation (Nakamura et al. 2013). The excessive production of extracellular proteases by *S. aureus* and the amplified generation of serine proteases by keratinocytes and metalloproteases in dermal fibroblasts could worsen the skin barrier damage. *S. aureus* disrupts the proteolytic balance in the skin (Williams et al. 2017). In severe strains, extracellular proteolytic activity was more pronounced than in less severe or healthy strains, suggesting that *S. aureus*’ ability to cause AD is strain-dependent. (Miedzobrodzki et al. 2002)*.* On the other hand, Commensal S. epidermidis produces lipoteichoic acid that prevents injury-induced TLR–3–3-mediated cutaneous inflammation via TLR-2 interaction (Nakatsuji and Gallo 2019). *S. epidermidis* regulates host cytotoxic and regulatory T lymphocytes for wound healing and immunological tolerance. *S. epidermidis* may increase keratinocyte antimicrobial peptide synthesis to fight infections in addition to its anti-inflammatory effects (Nakatsuji and Gallo 2019).

### Environmental and physiological variables

**Figure 2:**
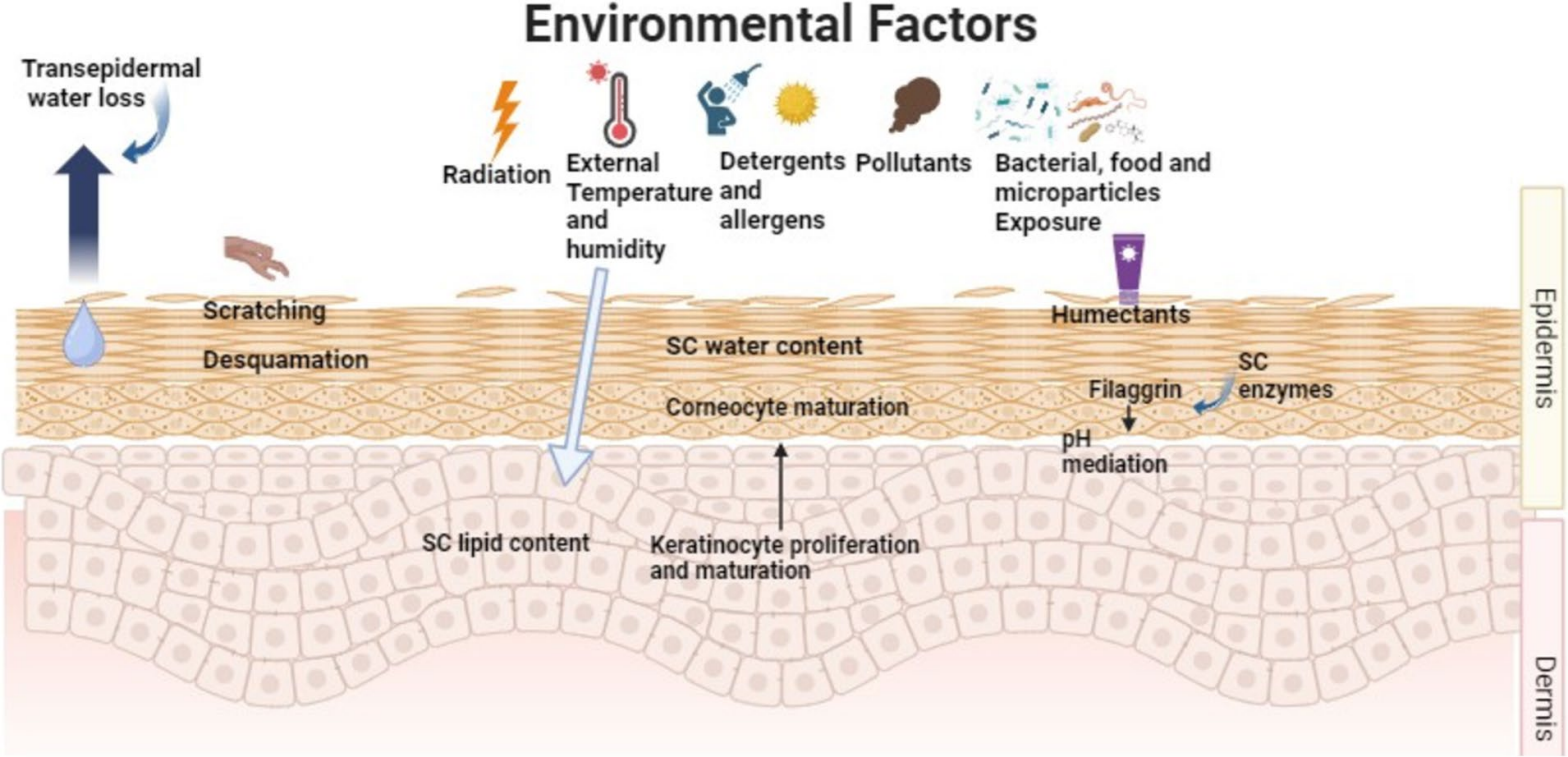
A demonstration of environmental and trigger factors that cause inflammation and the onset and severity of AD clinical course

The skin’s immune system, sensory organs, skin barrier, and microbiota can all be directly impacted by environmental variables such as UV radiation, pollutants, allergens, temperature, and humidity (Fig. 2). The “brick and mortar” model explains how anuclear corneocyte, or bricks, are placed in a lipid matrix that repels water, or mortar to, create an efficient barrier that prevents water loss and acts as a shield against allergens and noxious substances (Dijkhoff et al. 2020). Elevated UV exposure causes apoptosis, DNA damage, cutaneous inflammation, and dose-dependent degradation of the skin barrier. On the other hand, low exposure to UVB irradiation has advantageous anti-inflammatory, antipruritic, Staphylococcus aureus-inhibiting, and skin barrier-improving properties that may lower the risk of AD development. Similarly, temperature changes can cause cutaneous inflammation through channels known as the transient receptor potential family, which are involved in nociceptive, thermal, and mechanical perception. The activation of transient receptor potential vanilloid (TRPV) 1 in low temperatures might result in the release of proinflammatory cytokines and downregulate the expression of loricrin and filaggrin, ultimately causing skin barrier failure and pruritus (Engebretsen et al. 2016). Conversely, higher temperatures cause pruritus and proinflammatory cytokines to be produced, which in turn aggravates AD using TRPV 1, 3, and 4.

Undoubtedly, humanity is industrializing too quickly. Urbanization has several harmful effects, including air pollution. Toxins like particulate matter and volatile organic compounds from traffic-related air pollution and cigarette smoke disrupt the skin barrier, increase oxidative stress, induce proinflammatory signaling cascades, and cause dysbiosis. When paired with UV irradiation, ozone and air pollution increase inflammation. Sensitized people who are exposed to allergens more frequently, including pollen, experience skin inflammation as a result of T-helper (Th) cell 2 signaling. TEWL loss through the skin is influenced by environmental factors, such as humidity and temperature, in addition to deterioration of the skin barrier. An example of this would be a decrease in humidity and temperature, which would weaken the skin’s protective layer and make the skin more reactive to allergens and irritants, as well as mechanical stress.

Alcohol and cigarette smoking during pregnancy may increase the incidence of AD in children (Shaheen et al. 2014). The prevalence of AD increases with passive cigarette smoking exposure (Kantor et al. 2016). Previous studies have shown that maternal fish consumption during pregnancy reduces AD risk (Vaughn et al. 2019). The environment induces childhood atopic diseases, according to the “hygiene hypothesis”. Whereas, children raised in poor, rural nations with farm animals and unpasteurized milk are more likely to develop AD (Flohr et al. 2005). Herpes simplex virus and respiratory syncytial virus increase dermatitis risk (Martorano and Grayson 2018). In addition, early antibiotic exposure harms pregnant mothers and their newborns’ skin and gut microbiomes. Alternatively, probiotics enhance the microbiota (Lee et al. 2018). Research has shown that severe AD patients’ reduced skin barrier makes them more prone to food allergies, causing percutaneous food sensitivity (Papapostolou et al. 2022). Tests for common allergens can prevent allergies while safeguarding children’s nutrition (Oranges et al. 2015).

Physiological factors like skin pH (7.4) can prevent atopic dermatitis and maintain barrier function. By promoting barrier function and antimicrobial activity, the acidic milieu guards against atopic dermatitis (Ali and Yosipovitch 2013). Newborns have an acidic pH, whereas the elderly have a more basic pH owing to ceramide deficit and stratum corneum dryness; nonetheless (Oranges et al. 2015). Non-preventable problems can change skin pH; for instance, personal hygiene chemicals induce TEWL and skin injury by emulsifying skin lipids (Pelc et al. 2018).

Higher body mass index is linked to a marginally significant increase in the risk of AD. Based on information from 389,849 controls and 30,608 cases in the UK Biobank Resource, one group showed a marginally significant increase in AD risk of 1.02 for every kilogram/m^2^ increase in body mass index (Budu-Aggrey et al. 2021). High body mass index during early childhood increases the incidence of AD later in life (Manjunath and Silverberg 2022). One meta-analysis examining AD and coronavirus in 2019 (COVID-19) found that skin disorders like AD were linked to a higher risk of COVID-19 (Patrick et al. 2021).

Recent research has focused on the utilization of extant resources to enhance our comprehension of the genetic architecture of AD and to identify the variants that cause the disease. In this study, 30 genome-wide significant areas, including 5 novel loci, were successfully identified using biobank resources. This study emphasizes that various developing biobank datasets can be used to investigate genetic AD in the future (Sliz et al. 2022).

## Diagnosis and evaluation

Clinical phenotypes of AD vary widely according to age, severity, and ethnicity, reflecting a multifaceted and intricate interplay of features that could provide opportunities for preventive and therapeutic approaches (Patrick et al. 2021). Based on the age at which AD first manifests, pediatric patients with AD can be divided into three primary categories: very early onset, early onset, childhood-onset, and adolescent onset (Eichenfield et al. 2022). The severity of AD has been classified into three forms: mild, moderate, and severe (Hanifin et al. 2022). There are no approved diagnostic tests for AD; thus, the diagnosis is made primarily based on clinical observations and skin biopsies. In 87% of patients, pruritus is still the most common symptom and the initial indication of AD, resulting in pain, infections, anxiety, alteration of the skin barrier, and insomnia (Sidbury and Kodama 2018).

Children, teenagers, and adults develop rather diffuse flexural lesions that alternate between acute and chronic areas, in distinction to infants who frequently display acute eczematous pruritic lesions involving the face, cheeks, and torso (Schneider et al. 2021). It is advisable to carefully distinguish between possible diagnoses in elderly individuals because early-stage AD symptoms are comparable to those of psoriasis, erythroderma, scabies, and cutaneous T-cell lymphoma.

Precision medicine and society’s need to prevent and treat diseases cost-effectively have encouraged researchers to examine sensitization profiles or biomarkers that predict disease severity and identify individuals before clinical signs arise. The sensitization profiles of patients with AD can be examined by testing total immunoglobulin (IgE) and allergen-specific IgE against food and pollen allergens and estimating the ratio of specific to total IgE (Hu et al. 2020).

Cut-off points and scoring indices aid in assessing disease severity through identification and can aid in diagnosing, tracking progression, and evaluating treatments (Table 2). Measures like body surface area (BSA), the Investigator’s Global Assessment (e.g., V-IGA), the eczema area and severity index (EASI), the scoring atopic dermatitis (SCORAD), and the Patient-oriented eczema measure (POEM) are used to characterize a variety of disease severity indices (Futamura et al. 2016; Hanifin et al. 2022; Eichenfield et al. 2022). Patient-reported symptoms were recently found to be a top priority domain for the global, multi-professional harmonizing outcome measures for eczema (HOME) initiative. This consensus statement recommended the use of the POEM index, as well as an updated version of the SCORAD known as the patient-oriented SCORAD index and the EASI (Hanifin et al. 2022; Eichenfield et al. 2022). With these tools, physicians can accurately track the course of their patients’ diseases and tailor their care. A reduction of at least 75% of the baseline EASI score (EASI-75) is the main objective (Vestergaard et al. 2024).

**Table 2:** Diagnostic indices scale for atopic dermatitis severity diagnosis

Diagnostic index	Description of assessment	Advantages	Drawbacks
SCORAD Kunz et al. (1997); Schmitt et al. (2014)	The clinically validated scale measures disease extent using the rule of 9s, intensity using 6 clinical symptoms scored on a 4-point scale, and patient-reported pruritus and sleep loss.	Assesses BSA and lesion severity Sufficient validity, responsiveness, and interobserver reliability; Recommended by HOME	Its inter-observer reliability is uncertain Other non-AD variables can affect pruritus and sleep loss.
PO-SCORAD Stalder et al. 2011	Patient self-evaluation scale based on SCORAD criteria, optimized for patients and their families with illustrations and other evaluation support.	Assesses BSA and lesion severity Recommended by HOME Between clinic visits, patients can track AD symptoms.	Other non-AD variables can affect pruritus and sleep loss.
POEM Charman et al. (2013)	Validated score utilizing a 5-point rating system (up to 35) to evaluate 7 symptoms during the previous 7 days.	Designed for AD Scoring correlates with disease severity. Assess clinical trial populations appropriately. record symptoms frequency Practical for routine clinical practice Emerging in AD research and clinical trials	Good clinical utility but limited dermatology clinic utility does not quantify symptoms severity
IGA Futamura et al. 2016	Based on the morphological appearance of lesions, a global disease severity scale of 4 or 5 points is used.	Facilitates fast AD assessment	Non-standard ISGA definitions and diverse definitions in clinical study implementations.
BSA Eichenfield et al. 2022	Condition severity expressed as a percentage of overall BSA		Evaluation is difficult in patients with less severe lesions, especially with xerosis. Does not evaluate lesion severity
EASI Hanifin et al. (2022); Eichenfield et al. 2022	Validated scale using 7-point illness extent evaluation in 4 body regions, 4-point clinical sign severity, and 72-point maximum score.	Inter-observer reliability, internal consistency, validity, and responsiveness Recommended by HOME	Reduced sensitivity in low-BSA patients 6-minute assessment by a qualified investigator. Induration/papulation affects inter-evaluator variability. No evaluation of patient-reported symptoms
Health‐related quality of life (HrQoL) Thomas et al. (2021)	Dermatology life quality index (DLQI) for adults, children’s dermatology life quality index (CDLQI) for children and infant’s dermatology quality of life index (IDQoL) for infants. Includes 10 questions regarding the impact of AD on quality of life. A score of 0–30 indicates the highest level of QoL impairment.	Efficient for long term management	There is not a single QoL tool for AD that works for patients of all ages. further investigations required
Childhood atopic dermatitis impact scale (CADIS) Gabes et al. (2020); Thomas et al. (2021)	Instrument evaluating the QoL of AD-afflicted children under 6 and their families. Two dimensions with five domains explain 45 items each with five frequency responses. Scores are 0–180.	Excellent construct validity, test-retest reliability, internal consistency, and responsiveness Able to detect changes over time	Further investigations required

Adopted with modifications from Hanifin et al. (2022); Frazier and Bhardwaj (2020); Eichenfield et al. 2022

*AD* atopic dermatitis, *BSA* body surface area, *EASI* eczema area and severity index, *IGA* Investigator’s Global Assessment, *ISGA* Investigator’s Static Global Assessment, *POEM* patient-oriented eczema measure, *PO-SCORAD* patient-oriented scoring atopic dermatitis, *SCORAD* scoring atopic dermatitis

## Therapeutic directions for the management and treatment

### Medical interventions

**Figure 3:**
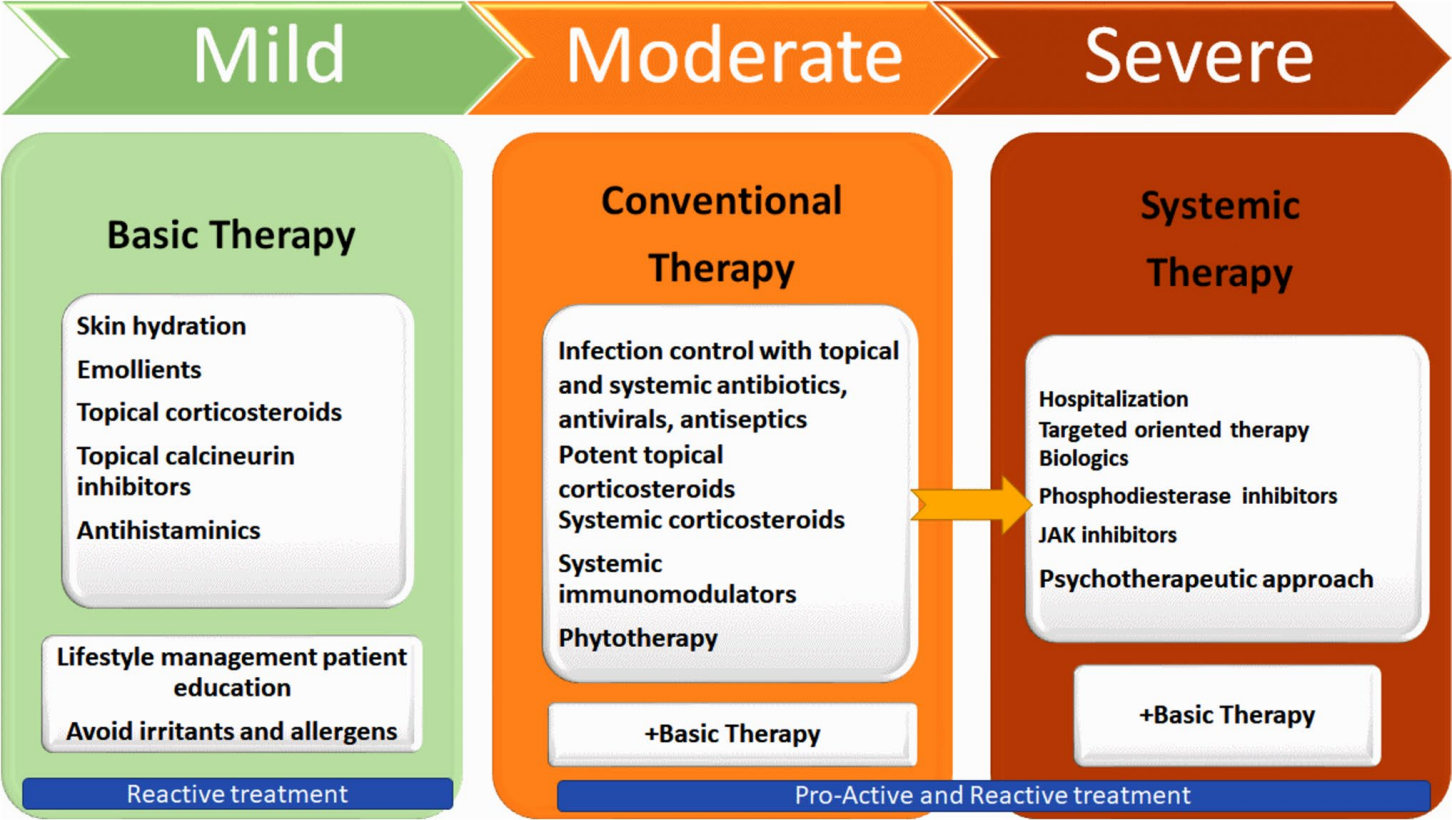
A diagrammatic representation of AD therapy  based on the severity of the condition

Figure 3 presents a diagrammatic representation of AD therapy based on disease severity.

#### Topical treatments

The European Task Force on Atopic Dermatitis (ETFAD) policy document recommends moisturizers for all AD patients (Vestergaard et al. 2024). Topical treatment is important for AD patients with BSA below 10%. Topical therapies include topical corticosteroids (TCS) and calcineurin inhibitors (TCI). Over 40 novel topical chemicals are being developed in a Phase 3 clinical study. Emerging medicines target the aryl hydrocarbon receptor (AhR), Janus kinase (JAK-receptors (licensed in the US as Ruxolitinib and in Japan as Delgocitinib), TRPV1, and phosphodiesterase-4 (PDE4) inhibitors (licensed in the US as Crisaborole) (Müller et al. 2024).

##### Topical corticosteroids (TCS)

Topical corticosteroids are the first-line treatment for AD, TCS reduces itching, inflammation, and redness by decreasing dendritic and Langerhans cell antigen processing and inflammatory cytokine levels (Calabrese et al. 2022). Hydrocortisone, triamcinolone, and betamethasone are commonly used for AD management. These drugs are used in creams, ointments, and lotions for ease of application. Hydrocortisone (1%) is the most effective for the face and neck, and clobetasol is the most effective for other skin areas (Kakkar et al. 2023). Although highly effective in treating AD, topical steroids can cause skin shrinkage, skin thinning, telangiectasia, and severe withdrawal when stopped. Secondary infections are also possible due to weakened immunity (Marshall et al. 2024). Children’s body surface area to weight ratio is higher than that of adults; hence, they experience a higher drug absorption rate. Indeed, many patients worry about the use of steroids during treatment. A recent study indicated that despite the enormous quantity of data supporting topical steroids for AD care, many patients are unwilling to use them (Marshall et al. 2024).

##### Topical calcineurin inhibitors (TCI)

Topical calcineurin inhibitors (TCI) are second-line and superior alternative approaches for AD management. TCIs are costly immunomodulators and are restricted to children aged > 2 years. There are two types of TCI: Elidel^®^ cream (pimecrolimus) at 1% for mild to moderate eczema and Protopic^®^ ointment (tacrolimus) at 0.1 or 0.3% for moderate to severe eczema. Calcineurin inhibitors suppress IL-2 synthesis, reducing T cell activation and immunological response (Abędź and Pawliczak 2019). TCI is usually applied twice daily in tender areas, such as the face and skin folds, where corticosteroids may cause skin atrophy or thinning (Eichenfield et al. 2014).

#### Systemic treatments

Although AD itch is mainly non-histamine, first- and second-generation antihistamines may be recommended for patients with trouble sleeping or scratching. Oral second-generation antihistamines, such as cetirizine or levocetirizine, are recommended for patients at work or school because first-generation antihistamines, such as diphenhydramine and hydroxyzine, are sedatives. Systemic immunosuppressive drugs such as, cyclosporine, azathioprine, and methotrexate are, administered when standard therapies fail. These medications are prescribed for treatment-resistant or severe AD. Cyclosporine is rather selective because it stops calcineurin from activating the nuclear factor of activated T cells (NFAT) (Vestergaard et al. 2024). Pretreatment renal and hepatic screening is recommended for systemic therapy candidates because these drugs can cause kidney or liver impairment (Kakkar et al. 2023).

#### New specific treatment approaches

Progress on new specific treatments has grown over the past few years (Table 3). The next section discusses these novel treatments.

**Table 3:** Recent biological and small molecules immunomodulators for AD therapy

Name	Route of delivery	Mechanism of action	Specific target	Current phase of clinical trials
Dupilumab	Injection	Cytokine antagonist	IL-4Rα	Approved for clinical use by EMA in 2017
Tralokinumab		Cytokine antagonist	IL-13	Approved for clinical use by EMA in 2021 ≥18 y (FDA) ≥12 y (EMA
Nemolizumab	Injection	Cytokine antagonist	IL-31	FDA initial approval in 2019 and reconfirmed 2023
Lebrikizumab	Injection	Cytokine antagonist	IL-13	EMA approved 2023, FDA approved 2024
Asivatrep	Topical	Cation channel antagonist	TRPV1	Phase 3 study
Tapinarof	Topical	Aryl hydrocarbon receptor modulator		FDA approved 2022, Not marketed yet
Crisaborole	Topical	Phosphodiesterase 4 inhibitor	regulating cyclic AMP	FDA approve in 2016
Abrocitinib	Oral	JAKi	JAK1	(FDA, EMA in 2022) ≥12 y
Baricitinib	Oral	JAKi	JAK1, JAK2	EMA-approved for adult AD 2020; in United States, only skin-related FDA approval is for adults with alopecia areata
Delgocitinib	Topical	JAKi	JAK1, JAK2, JAK3, TYK2	Japan approved 2020 ≥2 years
Ruxolitinib	Topical	JAKi	JAK1, JAK2	FDA approved 2021
Tofacitinib	Oral	JAKi	JAK1, JAK3	FDA approved 2017
Udapacitinib	Oral	JAKi	JAK1	FDA Approved2022

*FDA* Food and Drug Administration, *EMA* European Medicines Agency, *JAKI* janus kinase inhibitor

##### Biologicals

Comprehending the immune and genetic aspects of AD has aided identify targets for innovative treatments that more effectively target the disease’s underlying causes. Research and clinical trials have shown the practical value of biological therapy and have supported the development of patient-specific, targeted AD medicines (Caffarelli et al. 2023).

Dupilumab (Dupixent^®^) is an innovative US Food and Drug Administration (FDA)- and European Commission-approved therapy for children with moderate-to-severe AD aged 1 year and weighing at least 15 kg (D’Ippolito and Pisano 2018). It is an inhibitor of interleukin-4 (IL-4) receptor-α. This monoclonal antibody inhibits Janus kinase/signal transducers and activators of the transcription (JAK-STAT) signalling pathway by targeting the IL-4 and IL-13 signaling pathways. These cytokines are significant in the pathogenesis of atopic diseases. Dupilumab is usually injected subcutaneously every two weeks and is frequently used in combination with other treatments (D’Ippolito and Pisano 2018). Conjunctivitis is an adverse effect of dupilumab, and 28% of patients reported experiencing burning and a sensation of a foreign body in their eyes (Aszodi et al. 2019). SOLO 1 and SOLO 2 clinical studies involved enrolling individuals with moderate-to-severe atopic dermatitis whose condition was not well controlled by topical therapy in two randomized, placebo-controlled, phase 3 studies of the same design (Simpson et al. 2016). Patients were randomly randomized in a 1:1:1 ratio to receive subcutaneous dupilumab (300 mg) or placebo weekly or the same dose every other week alternating with placebo for 16 weeks. The primary outcome was the proportion of patients with a score of 0 or 1 (clear or almost clear) on the Investigator’s Global Assessment and a 2-point or more drop from baseline at week 16. SOLO 1 enrolled 671 patients and SOLO 2 708. In SOLO 1, the primary objective was achieved by individuals who got dupilumab every other week or weekly, compared to those who received placebo (*P* < 0.001). Similar results were observed in SOLO 2, with 36% of patients receiving dupilumab every other week or weekly experiencing the primary endpoint, compared to 8% of patients receiving placebo (*P* < 0.001). In the two trials, dupilumab significantly improved eczema area and severity index scores by at least 75% from baseline to week 16 compared to placebo (*P* < 0.001). Dupilumab also improved pruritus, anxiety, depression, and quality of life, pruritus, anxiety, and sadness. Dupilumab caused greater injection-site reactions and conjunctivitis than placebo.

To evaluate dupilumab’s long-term safety and effectiveness in AD patients. Adults who had previously taken part in phase 1 through 3 clinical trials of dupilumab for AD were assessed for long-term treatment in this multicenter, open-label extension study (NCT01949311). At the data cutoff (April 2016), this analysis looked at individuals who received 300 mg of dupilumab per week for up to 76 weeks. The main goal was safety, although efficacy was also assessed. The findings indicated that At cutoff, 92.9% of the 1491 enrolled patients (1042.9 patient-years) were undergoing therapy. There were no new safety signals, and the safety profile was in line with previously published trials (420.4 adverse events/100 patient-years and 8.5 serious adverse events/100 patient-years). Nasopharyngitis, conjunctivitis, and injection-site responses were among the most frequent adverse events. All efficacy endpoints, including measures of skin inflammation, pruritus, and quality of life, showed sustained improvement for up to 76 weeks. The study’s limitations include the absence of a control group, a small number of patients who received treatment for 76 weeks or more (median follow-up, 24 weeks), and patients who did not receive the recommended dosage schedule of 300 mg every two weeks. The study concluded that dupilumab’s use as a continuous, long-term therapy for patients with moderate to severe AD is supported by the safety and efficacy profile of this trial (Deleuran et al. 2020).

Health Canada, the FDA, and the European Commission has recently approved the use of talokinumab for patients with moderate-to-severe AD. This monoclonal antibody (IgG4λ) anti-IL-13, is fully humanized and competitively inhibits IL-13’s ability to bind to both the IL-13Rα1 and IL-13Rα2 receptor chains (Lytvyn and Gooderham 2023). With significant promise for efficacy and low toxicity, IL-13 downregulation is a prospective therapeutic target for AD (Lytvyn and Gooderham 2023). Patients with moderate-to-severe AD who have not responded to topical therapies are eligible for tralokinumab treatment provided they are 18 years of age or older. (Lytvyn and Gooderham 2023). For patients with moderate-to-severe AD who needed systemic medication, the ECZTRA 3 research planned a phase III trial to assess the safety and efficacy of tralokinumab in conjunction with TCS. Using a double-blind approach, patients were randomized in a 2:1 ratio to receive 300 mg of SC tralokinumab or a placebo every two weeks, plus a topical corticosteroid, if necessary, for 16 weeks. The percentage of patients who received tralokinumab at the end of the 16-week treatment period who had an IGA score of 0 or 1 and an EASI 75 was significantly higher in this experiment than in those who received a placebo. A sample of patients from both study arms was rerandomized to receive subcutaneous tralokinumab q2w or q4w for an extra 16 weeks after the initial treatment period. The majority of patients continued to respond favorably to treatment. When given tralokinumab q2w, 89.6% of patients maintained their IGA responses after 32 weeks. Furthermore, EASI 75 responses were maintained at 32 weeks in 92.5% of patients who took tralokinumab q2w. At 32 weeks, the IGA and EASI 75 responses were maintained in 77.6% and 90.8% of patients who took tralokinumab q4w, respectively (Ewulu et al. 2023).

A phase III trial called ECZTRA 7 assessed the safety and efficacy of tralokinumab plus TCS in persons with severe AD who were either contraindicated for cyclosporin A treatment or had previously experienced a failure with cyclosporin A treatment For 26 weeks, 277 adults in this multicenter, randomized, double-blind research got injections of either tralokinumab or a placebo every two weeks, in addition to TCS as needed. By week 16, more patients treated with tralokinumab plus TCS than those treated with placebo plus TCS had achieved the primary efficacy target of EASI 75. According to this trial, in persons with severe AD who are not responding to CyA therapy, tralokinumab plus TCS administered as needed significantly improves AD signs and symptoms. (Gutermuth et al. 2022).

The ECZTRA 5 trial was a randomized, double-blind, placebo-controlled phase II research that examined how tralokinumab affected vaccine immune response. In a 16-week study, 107 adult patients received tralokinumab 300 mg and 108 received a placebo q2w. At week 12, all patients received the Tdap and meningococcal vaccines, and the primary objectives were positive immunological responses to either vaccine at weeks 12 and 16. At week 16, the tralokinumab and placebo groups had similar immune responses to the Tdap (91.9% vs. 96.1%) and meningococcal (86.0% vs. 84.2%) vaccinations. This suggests that tralokinumab-treated persons with moderate-to-severe AD can continue treatment without interruption because routine non-live vaccinations are safe and effective (Merola et al. 2021). All reported studies showed no life-threatening AEs for tralokinumab. Upper respiratory tract infection, headache, somnolence, toothache, conjunctivitis, pruritus, pyrexia, nasopharyngitis, and acne were the most common systemic AEs. A localized injection site reaction with pain and erythema was described. The FDA and EMA have previously approved (2021) tralokinumab for the treatment of AD in adults; however, it is not currently authorized for use in adolescents.

Clinical trials have also examined the IL-31 monoclonal antibody nemolizumab. Among people with EASI ≥ 16 at baseline, nemolizumab therapy improved inflammation, pruritus, and sleep in a post hoc analysis of a phase 2b trial of moderate-to-severe AD (Silverberg et al. 2021). A 16-week, double-blind, phase 3 trial found that nemolizumab reduced AD pruritus more than placebo plus topical treatments (Kabashima et al. 2020).

Chen et al. (2019) investigated the in vivo effects of etokimab (ANB020), a monoclonal IgG antibody, in 12 patients with moderate to severe AD in a phase-2a study. At day 29, 83% of patients attained EASI-50 and 33% attained EASI-75 following a single systemic dosage of etokimab. In addition, a notable decrease in cutaneous neutrophil infiltration was observed compared with placebo. These findings suggest that suppressing IL-31 may be an additional part of AD therapy and may have a favorable impact on inflammatory responses (Chen et al. 2019). These findings led to the initiation of a Phase II study (NCT03533751).

Lebrikizumab is a humanized monoclonal antibody that binds soluble IL-13 and is highly selective in the non-receptor binding domain. lebrikizumab stimulates IL-13Rα2 to control endogenous IL-13 while inhibiting the IL-4Rα–IL-13Rα1 signaling complex (Moyle et al. 2019). A phase IIb trial examined the effectiveness, dose–response relationship, and safety of lebrikizumab monotherapy, with TCS utilized if needed, in 280 AD adults who failed topical treatment. Patients were randomized into four groups: 250 mg lebrikizumab with 125 mg q4w, 500 mg with 250 mg q4w, 500 mg at baseline and week 2 with 250 mg q2w, and placebo q2w. At week 16, all lebrikizumab groups showed a dose-dependent reduction in EASI scores compared to placebo, with significant differences seen as early as week 4. The lebrikizumab 250 mg group had higher response rates in secondary endpoints, such as IGA 0/1 EASI 50, EASI 75, and EASI 90. Placebo-treated patients needed three times more rescue TCS, started sooner and lasted longer, than lebrikizumab-treated patients (Guttman-Yassky et al. 2020). The ADore single-arm trial examined lebrikizumab’s safety and efficacy in adolescents with moderate-to-severe AD. Lebrikizumab was administered twice at 250 mg at week 1 and week 2, followed by a single injection q4w from weeks 4 to 52. Lebrikizumab improved skin clearance clinically from week 4 onward, with more patients improving over time. The study found that targeting IL-13 with lebrikizumab for moderate-to-severe AD in teenagers is promising (Paller et al. 2021). Similarly, other studies showed clinically significant improvements in all primary and secondary objectives (Blauvelt et al. 2021; Silverberg et al. 2023b). In all investigations, lebrikizumab demonstrated a good safety profile with no significant adverse events that could be fatal. Upper respiratory tract infections, nasopharyngitis, headaches, oral herpes, conjunctivitis, and coronavirus disease 2019 (COVID-19) were the most common systemic adverse events detected. There have been reports of injection site responses locally, with lebrikizumab groups experiencing frequency ranging from 1.3% to 5.7%. In November 2023, the EMA authorized lebrikizumab for use in AD; recently in 2024, the FDA has approved it.

Two more IL-13 inhibitors in the pipeline, eblasakimab and cendakimab, are presently being studied in phase II studies (NCT04800315). Eblasakimab is a monoclonal antibody that interferes with IL-13 and IL-4 signaling by targeting the IL-13Rα1 component of type 2 receptor. Eblasakimab was tested in AD patients in a phase Ib, double-blinded research. Preliminary data presented 52 participants randomized to receive eblasakimab at 200, 400, or 600 mg versus a placebo group. After 8 weeks, eblasakimab showed significant improvement over placebo. A decrease in EASI score was observed in all dose groups: 50% in 200 mg, 63% in 400 mg, and 61% in 600 mg, compared to 32% in the placebo group. Eblasakimab also increased EASI 50 and 75 responses compared to placebo. The peak PNRS score was lower in the 600 mg eblasakimab group compared to the placebo group. An analysis found that 400 and 600 mg eblasakimab dosages resulted in better clinical responses than 200 mg. At week 8, eblasakimab 600 mg significantly improved the mean percentage change in EASI, with more patients achieving EASI 50 and 75 compared to placebo. In addition, this study demonstrated that eblasakimab medication improved sleep disturbance and PNRS and patient-oriented eczema measure (POEM) ratings when compared to placebo. Pruritus, injection site erythema, and injection site edema were the most frequently reported adverse events (Veverka et al. 2024). Several other ongoing clinical trials are reported.

Humanized recombinant anti-IL-13 monoclonal antibody Cendakimab (RPC-4046). Cendakimab interacts to IL-13 at an epitope that overlaps with the IL-13Rα1 and IL-13Rα2 subunits, impeding their contact and signaling. A phase II multicenter, randomized, double-blind, parallel-group, placebo-controlled trial (NCT04800315) examined the efficacy and safety of three cendakimab dosage regimens in adults with moderate-to-severe AD. The study has four experimental groups. In group 1, participants received 720 mg of SC cendakimab weekly (qw) for 16 weeks. In group 2, subjects received SC cendakimab 720 mg q2w and placebo alternatively. Group 3 participants received SC cendakimab 360 mg q2w and a placebo weekly. Placebo subjects got SC placebo qw. The study’s main endpoint was the mean percentage change in EASI scores from baseline to week 16. In the trial, cendakimab 720 mg qw resulted in a higher drop in EASI score (-84.41%) compared to the 720 mg q2w, 360 mg q2w, and placebo groups (-76.03, 78.93, and 62.7%, respectively). In the cendakimab 360 mg q2w group, 38.2% of patients attained an IGA score of 0 or 1, compared to 33.3% in the 720 mg qw group and 24.4% in the 720 mg q2w group. The most common adverse effects in this study were upper respiratory tract infection, allergic conjunctivitis, COVID-19, and headache (Blauvelt et al. 2024). Even though cendakimab passed its phase II trial, development has stopped. After meeting the primary goal, cendakimab’s manufacturer observed that AD treatment is extremely competitive and that it does not offer a significant advantage over other medications (Teixeira et al. 2024). Table 4 presents clinical trials that investigated new biologics for the treatment of atopic dermatitis (AD)

**Table 4:** Summary of clinical studies on new biologics and small molecules for atopic dermatitis (AD), including their mechanisms of action, side effects, and conclusions

Therapy	Mechanism of Action	Clinical Study Findings	Side Effects	Conclusion	Reference
Dupilumab	Monoclonal antibody targeting IL-4Rα, inhibiting IL-4 and IL-13 signaling pathways	Demonstrated significant improvement in AD severity and quality of life in multiple clinical trials.	Conjunctivitis, injection site reactions, nasopharyngitis.	Effective in reducing AD symptoms with a favorable safety profile.	Eichenfield et al. (2021)
Lebrikizumab	Monoclonal antibody targeting IL-13	Phase III trials showed significant improvement in AD severity compared to placebo.	Upper respiratory tract infections, headache, injection site reactions.	Promising efficacy in AD treatment with manageable side effects.	Eichenfield et al. (2021)
Tralokinumab	Monoclonal antibody targeting IL-13	Demonstrated significant improvement in AD severity in clinical trials.	Upper respiratory tract infections, conjunctivitis, injection site reactions.	Effective in reducing AD symptoms with a favorable safety profile.	Eichenfield et al. (2021)
Upadacitinib	Janus kinase (JAK) inhibitor, modulating multiple cytokine signaling pathways	Phase III trials showed significant improvement in AD severity compared to placebo.	Acne, nasopharyngitis, headache, increased risk of infections.	Effective in reducing AD symptoms; requires monitoring for infections.	Butala et al. (2023)
Abrocitinib	JAK1 inhibitor, modulating multiple cytokine signaling pathways	Demonstrated significant improvement in AD severity in clinical trials.	Nausea, headache, nasopharyngitis, increased risk of infections.	Effective in reducing AD symptoms; requires monitoring for infections.	Butala et al. (2023)
Nemolizumab	Monoclonal antibody targeting IL-31 receptor A, inhibiting IL-31 signaling	Phase III trials showed significant improvement in pruritus and AD severity compared to placebo.	Nasopharyngitis, upper respiratory tract infections, peripheral edema.	Promising efficacy in reducing pruritus and AD severity with manageable side effects.	Eichenfield et al. (2021)
Baricitinib	JAK1/JAK2 inhibitor, modulating multiple cytokine signaling pathways	Demonstrated significant improvement in AD severity in clinical trials.	Upper respiratory tract infections, headache, increased risk of infections.	Effective in reducing AD symptoms; requires monitoring for infections.	Butala et al. (2023)
Ebglyss	Monoclonal antibody targeting IL-13	Clinical trials involving over 1,000 patients with moderate-to-severe eczema demonstrated the drug's efficacy, particularly for those not responding to topical or systemic treatments.	Not specified in the provided source.	Approved by the U.S. FDA for use in adults and children over 12 years old; offers a once-monthly dosing option.	Teixeira et al. (2024)

##### Small molecules

Compared to biologics, small molecules (<0.5 kDa) intreat more frequent dosing and produce more off-target effects when administered systemically. Phosphodiesterase-4 (PDE-4) and Janus kinase (JAK) inhibitors represent new therapeutic options for targeting the underlying inflammatory processes in AD (Kamata and Tada 2023).

An innovative approach to managing inflammatory and immunological disorders is using Janus kinase inhibitors (JKI). Phosphotransferases known as Janus kinases (JK) are activated enzymatically when cytokines bind to their receptors (Berbert Ferreira et al. 2020). JAK inhibitors target Janus kinases, which are important signaling pathways that connect different cytokine receptors to STAT transcription factors and are involved in cytokine generation. Since this signaling pathway provides the basis for the expression of IL-2, IL-4, and numerous other cytokines, JAK inhibitors offer a promising treatment for AD (Ahluwalia et al. 2017). Different etiological processes, including hematopoiesis, immunological fitness, tissue repair, inflammation, apoptosis, and adipogenesis, are mediated by the JAK-STAT downstream signaling pathway and are linked to a range of diseases and pathological behaviors (Maji et al. 2024). Different JAK inhibitors have different selectivity, degree of inhibition, and safety characteristics (Milakovic and Gooderham 2021). JAKi have the potential to revolutionize the treatment of some people with AD, and their benefit–risk ratio appears to be reasonable in this patient population (Schwartz et al. 2018).

For AD in adults and children aged more than two years, Difamilast is licensed in certain countries (Saeki et al. 2022). There were significant differences between the difamilast and placebo groups (18.1%) in a 4-week phase III research in children with mild-to-moderate AD. There is an ongoing phase III trial for infants older than three months to 2 years. Phase III trials involving 0.15% roflumilast cream in adults and children ≥6 years of age have produced encouraging preliminary data. A three-year-old phase III trial is now occurring (Saeki et al. 2022).

Baricitinib is a first-in-class oral JAK1/JAK2 inhibitor approved in 2020 by the European Medicines Agency for adults ≥ 18 years for moderate-to-severe AD however, is not yet been FDA-approved for AD in the United States. In clinical trials. baricitinib was superior to the placebo group (Yim et al. 2024). In all groups, nasopharyngitis, herpes simplex, headache, and back discomfort were the most frequent adverse events (≥5%). Pediatric investigations are ongoing (Bieber et al. 2021; Yim et al. 2024).

After topical and oral adminstration, the first-generation, non-selective JAK inhibitor delgocitinib (JTE-052/LEO 242549) improved allergic contact sensitization and D-like inflammation in animal models (Yamamoto et al. 2020). After phase III studies, Japan approved topical delgocitinib ointment for moderate to severe AD in 2020 (Nakagawa et al. 2020; Chovatiya and Paller 2021). However, cream development for chronic hand eczema (LEO 24249) has been discontinued in the United States and Europe (Chovatiya and Paller 2021).

In 2021, ruxolitinib, a JAK1/JAK2 JAKi 1.5% cream, received FDA approval for the treatment of mild-to-moderate AD ≥12 years. It has not been a widely used component of the traditional therapy arsenal (Yim et al. 2024). Ruxolitinib’s 8-week efficacy in the Investigator’s Global Assessment (IGA) and itch reduction compared to the vehicle were reported in two phase 3 trials (Papp et al. 2021; Papapostolou et al. 2022). The critical investigation revealed that the treatment was effective and tolerated for itch alleviation. Nasopharyngitis is the most prevalent side effect (Kleinman et al. 2022).

Tofacitinib, a first-generation small molecule JAK1/3 inhibitor, the FDA in November 2012 approved as a 5 mg twice daily (bid) oral dosage for moderate-to-severe rheumatoid arthritis in adults. Topical Tofacitinib was tested for mild-to-moderate AD. IN A 4-week, phase 2a randomized double-blinded vehicle-controlled trial (RDBVCT) in adults 18 to 60 years old, Tofacitinib 2% ointment compared to vehicle satisfied the main endpoint of percentage change from baseline eczema area and severity index (%EASI) at week 4 (81.7% vs 29.9%) (Bissonnette et al. 2016). Tofacitinib 2% ointment achieved secondary endpoints such as improved IGA scores (IGA 0/1) and %IGA, %BSA, and %itch-NRS, detected as early as day. Nasopharyngitis and urinary tract infections were the most common treatment-emergent adverse events (Chovatiya and Paller 2021). However, topical tofacitinib is not yet commercialized.

Second-generation JAK inhibitors, such as upadacitinib and abrocitinib, have been developed in the pursuit of JAK1-selective drugs, which are crucial for the activity of multiple cytokines implicated in AD. These agents have demonstrated rapid onset of action and high efficacy in clinical trials showing superior efficacy compared to some established treatments. Both have been approved by the European Commission, Health Canada, and the FDA for use in moderate-to-severe AD patients aged 12 years and older (Lytvyn and Gooderham 2023). In particular, JAK1-selective inhibitor SHR0302 has been developed for oral use in moderate-to-severe AD and topical application in mild-to-moderate AD. (Lin et al. 2020). In a phase III study, upadacitinib (approved in 2019 for rheumatoid arthritis) and abrocitinib improved patient outcomes and pruritus severity. (Blauvelt et al. 2021). Upadacitinib has shown promising results in long-term studies. Data indicate that upadacitinib maintains its efficacy in reducing AD symptoms over prolonged use, with an acceptable benefit-risk profile. Common adverse events include acne, nasopharyngitis, and headache, with serious adverse events being infrequent. Abrocitinib has been evaluated in long-term studies, demonstrating sustained efficacy in symptom reduction. The safety profile remains consistent with short-term studies, with nausea, headache, and nasopharyngitis among the most reported adverse events. Serious adverse events are rare but necessitate monitoring. Additionally, abrocitinib reduced itching better than dupilumab (Bieber et al. 2021; Lytvyn and Gooderham 2023).

Cerdulatinib’s combined targeting of JAKs and SYK is a promising topical pharmaceutical intervention. SYK is a crucial signaling protein for the FcεRI receptor on epidermal Langerhans cells, inflammatory dendritic cell count, and T-cell activation (Klaeschen et al. 2021). It was also reported that Cerdulatinib gel reduced epidermal thickness, inflammatory dendritic cells, and transcriptome inflammatory signatures in mild to moderate AD in a phase Ib study (Kim et al. 2020). This phase 1b, single-center, double-blind trial examined the safety and efficacy of cerdulatinib gel 0.37% and its effects on lesional (LS) skin molecular profiles in individuals with mild-to-moderate AD. Mild-to-moderate AD patients (*N* = 10) were randomized 4:1 to twice-daily topical cerdulatinib gel 0.37% or vehicle for 14 days. In the cerdulatinib group (*n* = 8; female = 4), the mean age was 30.8 years, baseline mean (SD) eczema area and severity index (EASI) was 4.0 (2.0), body surface area affected was 4.3% (2.0), and pruritus numeric rating scale score was 4.4 (1.8). In the vehicle group (*n* = 2; female = 2), mean age was 23.5 years, baseline mean (SD) EASI was 2.4 (0.8), body surface area affected was 3.0% (0.0), and pruritus numeric rating scale score was 1.5 (2.1). Eight of 10 individuals had 35 treatment-emergent adverse events, all mild (34 of 35; 97%) or moderate (1 of 35; 3%). Cerdulatinib’s most prevalent treatment-emergent adverse event was headache, which affected 3 of 8 participants. There were no clinically significant impacts on vital signs and electrocardiograms, and no patients quit treatment due to adverse events (Piscitelli et al. 2021).

Gusacitinib selectively inhibits JAK and SYK, which are tyrosine kinases activated by proinflammatory cytokines such IL-1β and IL-17. Gusacitinib appeared to be safe and well-tolerated in an early phase 1b study (RDBPCT). Only the highest dosages of gusacitinib met the EASI-50 secondary objectives and baseline itch-NRS improvement (Bissonnette et al. 2019). A separate phase 2b study (study to evaluate ASN002 in subjects with moderate to severe chronic hand eczema; NCT03728504) showed promising efficacy and safety (Rodenbeck et al. 2016). A double-blind, placebo-controlled, multicenter, phase 2 trial was conducted to assess the safety and effectiveness of gusacitinib (Jimenez et al. 2023). For 12 weeks, 97 patients with chronic hand eczema were randomized (1:1:1) to either a placebo or gusacitinib (40 or 80 mg) (part A). Gusacitinib was then administered to the patients during part B (week 32). At week 16, the modified total lesion-symptom score decreased by 69.5% (*P* < 0.005) for patients taking 80 mg of gusacitinib, compared to 49.0% for those taking 40 mg (*P* = 0.132) and 33.5% for those taking a placebo. Physician’s Global Assessment significantly improved in 31.3% of patients who took 80 mg compared to 6.3% who took a placebo (*P* < 0.05). Patients who received 80 mg saw a 73.3% drop in the hand eczema severity index compared to those who received a placebo (21.7%) (*P* < 0.001). Hand discomfort significantly decreased in those taking 80 mg (*P* < 0.05). Significant decreases in the modified total lesion-symptom score (*P* < 0.005), Physician’s Global Assessment (*P* = 0.04), and hand eczema severity index (*P* < 0.01) compared to placebo were noted as early as week 2 (80 mg gusacitinib). Nasopharyngitis, headache, nausea, and upper respiratory infections were among the side effects. The FDA granted gusacitinib quick-track designation in February 2021 for the treatment of moderate-to-severe chronic hand eczema.

##### Phototherapy

In resistant human AD, phototherapy is one of several therapies that can successfully reduce inflammatory skin lesions without having systemic side effects (Rodenbeck et al. 2016). The advantages of narrow-band ultraviolet B (UVB) phototherapy include high efficacy, comfort, accessibility, and minimal danger. The joint task force (JTF) recommends photochemotherapy with psoralen and UVA for severe and extensive AD symptoms, UVB for chronic AD, and UVA for acute exacerbations. The dosing and frequency of phototherapy depend on the lowest erythema dose, Fitzpatrick skin type, or both (Nguyen et al. 2023). When using phototherapy in children with AD, care is advised due to inadequate data. The effectiveness of this treatment has been demonstrated in numerous human skin conditions, including vitiligo and plaque psoriasis, and its few known adverse effects make it safe to use (Rodenbeck et al. 2016). In recent studies, excimer light therapy was successfully applied to human patients with AD (Kurosaki et al. 2020).

##### Ongoing trials

Numerous immune-mediated inflammatory illnesses have been examined modulating the sphingosine-1-phosphate receptor (S1P). Etrasimod is an oral selective, modulator of S1P receptors 1, 4, and 5 that lowers dermatitis and the number of lymphocytes drawn to an inflammatory location (Silverberg et al. 2023a, b). Measured from baseline to week 12 in 140 patients, the phase 2 trial found that the percent decrease in EASI score was −57.2% in the etrasimod 2 mg group and −48.4% in the placebo group (*P* = 0.18). Even though the trial’s main goal was not achieved, certain scores determined by the doctors and patients were significant. Patients with moderate-to-severe AD who have not responded to prior treatments are now being recruited for a phase 2/3 trial for further investigation due to the treatment’s safety and clinical efficacy (NCT05732454) (Silverberg et al. 2023a).

The aryl hydrocarbon receptor (AhR) agonist Tapinarof cream 1% (VTAMA^®^) was developed as a once-daily topical treatment for atopic dermatitis and plaque psoriasis by Dermavant Sciences Inc., a subsidiary of Roivant Sciences Inc. AhR is a transcription factor that is dependent on ligands and plays a part in immune-mediated skin responses downregulating Th17 and Th2 cytokines involved in plaque psoriasis and atopic dermatitis, respectively. Resulted in skin barrier normalization (by increasing the expression of keratinocyte-associated proteins like filaggrin, loricrin, and involucrin) and reduced oxidative stress (Bissonnette et al. 2023). The USA authorized 1% tapinarof cream in May 2022 for topical treatment of adult plaque psoriasis (https://www.dermavant.com/). A 12-week, double-blind, randomized Phase 2b trial in adolescents and adults with AD treated with 0.5% or 1 % tapinarof cream or vehicle improved eczematous lesions and itch. Side effects included nasopharyngitis, upper respiratory tract infection, AD worsening, and folliculitis (Paller et al. 2021). This innovative treatment is presently in phase 3 treatment of atopic dermatitis (Bissonnette et al. 2023; Müller et al. 2024).

A phase 2 trial was conducted to investigate the antimicrobial peptide omiganan, which is active against *S. aureus*. No clinical improvement was noted, despite reductions in *S. aureus* load and dysbiosis observed in all omiganan treatment groups (Niemeyer–van Der Kolk et al. 2022). Although omeganan’s efficacy as a monotherapy is limited, its potential as an adjuvant therapy may increase with a deeper understanding of the microbiome’s function. As of right now, no *S. aureus* vaccination has been authorized. Nonetheless, strategies focusing on *S. aureus* toxins are being developed. Probiotics used topically appear to be a promising treatment option for AD-causing *S. aureus*.

Several novel AD medications target T2-centered immune differentiation caused by OX40L binding to OX40. OX40 and its ligand OX40L, TNF-receptors, are essential mediators of T-cell activation, clonal expansion, survival, memory cell formation, and apoptosis suppression. Lesional cutaneous T cells in AD Patients express OX40. Two antibodies that block OX40 and OX40L are, rocatinlimab and amlitelimab (Ferrara et al. 2024). At the end of a phase 2 trial in (2022), rocatinlimab (KHK4083) exhibited a better percent change in EASI from baseline compared to placebo (*P* < 0.001). (NCT03703102) (Guttman-Yassky et al. 2023). Subcutaneous rocatinlimab (AMG451/KHK4083) is in Phase 3 trials for moderate-to-severe AD in adults.

Building on the success of antimicrobial peptide topicals, live biotherapeutic products (LBPs) represent a new and emerging class of topical medicine. Leveraging living bacteria and fungi has been shown to ameliorate microbial dysbiosis early on and result in clinically significant changes in AD (Vargason and Anselmo 2021).

Anti-transient receptor potential vanilloid (TRPV1), a nonselective cation channel, is found in mast cells, cutaneous sensory neurons, and keratinocytes, controls inflammation and, mediates histamine-dependent and -independent itch by releasing central neuropeptides such as substance P (SP) and calcitonin gene-related peptide (Schuler et al. 2023). Asivatrep is an antagonist of transient receptor potential vanilloid subfamily V member 1 (TRPV1). In the phase 3 trial, subjects with mild-to-moderate AD aged 12 or older who received twice-daily application of asivatrep1.0%, (TRPV1) showed, considerable improvements in AD symptoms by the eighth week compared with the placebo group (Park et al. 2022). In an 8-week phase III study comprising patients aged >12 years with mild-to-moderate AD, the primary endpoint, IGA (0, 1), was achieved by 36.0% in the 1.0% asivatrep cream group and 12.8% in the vehicle group (*P* < 0.001) (Park et al. 2022).

Phosphodiesterase-4 (PDE4) plays a pivotal role in the production cytokines of IL-4 and IL-13. These inhibitors prevents the degradation of cyclic adenosine monophosphate (cAMP) by phosphodiesterase, which typically lowers inflammation (Yang et al. 2019). By downregulating PDE-4, cAMP increases lead to downregulation of NFκB, an important regulator of cytokine production (e.g., of IL-4, -5, -10). The topical phosphodiesterase-4 inhibitor (PDE4i), Crisaborole (2% ointment twice a day), was approved by U.S. Food and Drug Administration (FDA) in 2016, for the treatment of mild to moderate AD in patients aged 3 months and older (Ferrara et al. 2024). Crisaborole outperformed placebo in the investigator’s Static Global Assessment (ISGA) success in mild-to-moderate AD children aged 2–17 (Luger et al. 2022).

A gram-negative bacterium called Roseomonas mucosa was reported to improve AD and lower the burden of *S. aureus* in both adults and children with AD in preclinical research (Myles et al. 2018). The, *S. aureus* load, topical steroid dosage, and illness severity were all significantly reduced after receiving *R. mucosa* treatment. Treatment-related side effects were not observed.

It was discovered that certain strains of *S. hominis* produce an autoinducing peptide that inhibits the regulatory quorum sensing mechanism of *S. aureus* accessory genes and stops the bacteria from forming biofilms (Williams et al. 2019).

Imbalances in the endogenous opioid pathway may contribute to chronic itch. Therapies targeting mu (μ) opioid receptors, kappa(κ)-opioid receptors, or both have been beneficial in treating persistent itch, especially uremic and cholestatic itch (Phan et al. 2012). The κ-opioid-R-agonist difelikefalin is licensed for moderate-to-severe pruritus in individuals requiring haemodialysis, and is being evaluated for moderate-to-severe itch in patients with AD (Deeks 2021).

##### Comparative efficacy and safety and clinical considerations

In summary, the integration of targeted biologics and small-molecule inhibitors into AD treatment has enhanced therapeutic outcomes by specifically modulating key inflammatory pathways, offering improved efficacy and safety profiles compared to traditional therapies. Traditional systemic therapies, such as cyclosporine and methotrexate, have been used for decades in AD management. While effective in the short term, their long-term use is often limited by safety concerns, including nephrotoxicity for cyclosporine and hepatotoxicity for methotrexate. These adverse effects necessitate regular monitoring and may restrict prolonged use (Eichenfield et al. 2022). Emerging therapies like dupilumab, upadacitinib, and abrocitinib offer targeted mechanisms of action, potentially leading to improved long-term safety profiles compared to traditional systemic treatments. Table 4 presents an overview of various therapies, their mechanisms, clinical findings, side effects, and conclusions based on recent studies and reviews.

Although dupilumab and eblasakimab target the IL-4 and IL-13 pathways, their modes of action are different. Eblasakimab inhibits the development of the IL-13Rα1/IL-4Rα heterodimer receptor signaling complex by binding specifically to the IL-13Rα1 subunit of the type 2 receptor. By protecting the type 1 receptor and blocking only IL-4 and IL-13 signaling through the type 2 receptor, this strategy may lessen the unintended consequences of broad cytokine inhibition. By targeting the α subunit in the IL-4 receptor, dupilumab, a completely human immunoglobulin 4κ monoclonal antibody, blocks IL-4 and IL-13 signaling through the type 2 receptor (IL-4Rα/IL-13Rα1) and inhibits IL-4 signaling via the type 1 receptor (IL-4Rα/common γ chain). The immune response may be impacted more broadly as a result of this wider suppression. Compared to the more comprehensive inhibition obtained by completely blocking the IL-4 receptor with dupilumab, eblasakimab’s selective targeting of the type 2 receptor may provide benefits in regulating Th2 cytokines without causing an increase in Th1 cytokines(Moyle et al. 2019).

In clinical trials and real-world investigations, conjunctivitis has been recorded in as many as 25% of patients, making it a significant adverse event linked to the use of dupilumab. It is unclear exactly what mechanism causes this phenomenon. Dupilumab, on the other hand, may trigger a Th1 response by targeting IL-4, which could result in reduced mucin synthesis and interferon γ-mediated goblet cell death, which would cause conjunctivitis and dry eyes (Ferreira and Torres 2020).

The clinical IL-13 monoclonal antibodies cendakimab, tralokinumab, and lebrikizumab were examined for their cell-based functional activity and in vitro binding affinities. Lebrikizumab was found to have a greater binding affinity to glycosylated and aglycosylated versions of IL-13 than cendakimab and tralokinumab. A slower dissociation rate is what drives this higher binding affinity. Lebrikizumab was more effective than tralokinumab and cendakimab at blocking the effects of IL-13 in cell-based neutralization tests. 11. Moreover, unlike cendakimab and tralokinumab, lebrikizumab binds to a unique epitope on IL-13 that does not prevent IL-13 from being naturally cleared through the IL-13Rα2 receptor. These in vitro results demonstrate the molecular and mechanistic variations between cendakimab, tralokinumab, and lebrikizumab, which could result in varying clinical efficacy for AD patients (Okragly et al. 2023).

Continuous monitoring for adverse events remains essential, especially concerning the risk of infections and other immune-related conditions associated with JAK inhibitors. When selecting a treatment modality, factors such as patient age, comorbidities, and disease severity should guide decision-making. Biologics may be preferred for their favorable safety profile, especially in patients with contraindications to immunosuppressive therapies. Conversely, JAK inhibitors might be considered for patients requiring rapid disease control, with careful monitoring for potential adverse effects (Paller et al. 2021; Butala et al. 2023; Teixeira et al. 2024). It is worth noting that, the lack of head-to-head research and the varying designs of individual studies make it difficult to make direct comparisons between various IL-13 inhibitors or between IL-13 inhibitors and other medications, such as dupilumab and Janus kinase inhibitors. To determine the role of selective IL-13 inhibitors in the treatment of AD, this knowledge gap must be filled in further trials and network meta-analyses. In summary, Table 5 provides a comparative overview of various treatments for atopic dermatitis, highlighting their administration routes, mechanisms of action, efficacy, safety considerations, and FDA-approved products.

**Table 5:** A comparative overview of selected treatments for atopic dermatitis (AD), including traditional medications and newer therapies

Treatment	Administration	Mechanism of action	Efficacy	Safety considerations	FDA-approved products
Topical corticosteroids	Topical	Anti-inflammatory; suppresses cytokine production and inflammatory cell migration.	Effective for mild to moderate AD; rapid symptom relief.	Skin atrophy, striae, telangiectasia with prolonged use; potential systemic absorption, especially in children.	Hydrocortisone, betamethasone, clobetasol.
Topical calcineurin inhibitors	Topical	Inhibits calcineurin, reducing T-cell activation and cytokine release.	Effective for mild to moderate AD; suitable for sensitive skin areas.	Burning sensation at application site; potential increased risk of lymphoma and skin malignancies (black box warning).	Tacrolimus (Protopic), pimecrolimus (Elidel).
Topical PDE4 inhibitors	Topical	Inhibits phosphodiesterase 4, leading to increased intracellular cAMP and reduced inflammatory cytokine production.	Effective for mild to moderate AD; improvement in pruritus and lesion severity.	Application site pain; potential for hypersensitivity reactions.	Crisaborole (Eucrisa).
Topical JAK inhibitors	Topical	Inhibits Janus kinase enzymes, interfering with cytokine signaling involved in the inflammatory process.	Effective for mild to moderate AD; reduction in itch and inflammation.	Application site reactions; potential risk of systemic absorption and immunosuppression.	Ruxolitinib (Opzelura).
Oral immunosuppressants	Oral	General immunosuppression; inhibits DNA synthesis and cell proliferation.	Effective for severe AD; used when topical treatments are inadequate.	Hepatotoxicity, nephrotoxicity, bone marrow suppression; increased risk of infections and malignancies.	Methotrexate, cyclosporine, azathioprine.
Biologics (e.g., Dupilumab)	Subcutaneous	Monoclonal antibody targeting IL-4 receptor α, inhibiting IL-4 and IL-13 signaling pathways.	Effective for moderate to severe AD; significant improvement in clinical symptoms and quality of life.	Conjunctivitis, injection site reactions; potential for systemic hypersensitivity reactions.	Dupilumab (Dupixent).
Oral JAK inhibitors	Oral	Inhibits Janus kinase enzymes, modulating multiple cytokine signaling pathways involved in the inflammatory process.	Effective for moderate to severe AD; rapid improvement in symptoms.	Increased risk of infections, including serious infections; potential for thrombosis and malignancies; requires regular monitoring.	Upadacitinib (Rinvoq), abrocitinib (Cibinqo).

### Nonmedical interventions and lifestyle adjustments

Nonmedical interventions are needed to address the triggers and contributing factors that aggravate AD symptoms. Nonmedical treatments emphasize skincare and lifestyle improvements. The skin’s protective barrier can be kept by avoiding harsh cleansers, hot water, and vigorous rubbing. Fragrance-free, hypoallergenic moisturizers and gentle non-soap cleansers keep the skin nourished and prevent flare-ups. Lifestyle adjustments can reduce stress, allergies, and irritants. Patients with AD can improve their quality of life by combining pharmacological therapy with nutritional changes and stress-reduction measures such as meditation or yoga. (Calabrese et al. 2022). Behavioral and psychological support are crucial to AD patients and their families. They help patients understand and break the cycle of itching and scratching by identifying behavioral and emotional triggers, and stress management (Sanders and Akiyama 2018). Children with AD can benefit from being diverted and redirected to useful tasks like moisturizing, coloring or painting, or squeezing a stress ball. Children-friendly relaxation and sleep applications can assist in AD treatment. Psychologists can assist AD patients in gaining confidence and accepting adjustments; however, further study is needed to determine their effects on AD management (Kakkar et al. 2023).

Food allergies comprise 35% of babies and children with moderate-to-severe dermatitis, making qualified dieticians necessary for multidisciplinary AD therapy. (Mehta and Fulmali 2022). Children with food allergies benefit from dietary counseling regarding energy, weight, length/height, and vitamin intake (Hermes et al. 2022).

## Artificial intelligence and prospects for AD

The last ten years saw the first practical examples of artificial neural networks (ANNs) being used in artificial intelligence (AI), as it has already highlighted the significance of applying data-driven approaches to AD etiopathogenesis (Maulana et al. 2023a, b). Two popular AI techniques are machine learning (ML) and deep learning (DL), with DL performing more effectively when handling complicated features and huge datasets (Li Pomi et al. 2024). Computer-aided diagnosis (CAD) solutions and convolutional neural networks (CNN) have been applied specifically in dermatology to improve picture categorization, object detection, and other analytical procedures (Li et al. 2022). AI has progressively impacted precision treatment for such diseases, using unsupervised ML techniques to cluster AD patients based on cytokine and chemokine expression profiles to enable endotypic diagnosis of AD and other allergic conditions (Yamamoto et al. 2020). Recent breakthroughs in this field include an ML classifier that uses intestinal epithelial transcriptome and intestinal microbiome data to reliably and automatically detect AD and identify relevant novel biomarkers (Jiang et al. 2022). In addition, the combination of advanced ML algorithms and confocal Raman microspectroscopy helps uncover clinically significant skin chemical indicators that distinguish AD patients from healthy controls (Dev et al. 2022). The results of training CNNs to recognize living cells using multiphoton tomography (MPT) imaging are promising. (Li Pomi et al. 2024).

Meanwhile, Wu’s group, which had proposed a DL-based AI dermatology diagnosis assistant (AIDDA), had achieved similar encouraging outcomes, with a 92.57% accuracy rate for AD diagnosis, 94.41% specificity, and 94.56% sensitivity (Wu et al. 2023). Medela et al. (2022; Wu et al. (2023) used the Deep Expectation technique on 604 dermatological images to achieve an 84.6% diagnosis accuracy. Al-masni et al. (2018) observed 77.11% testing accuracy for melanoma pictures using full-resolution CNN. In terms of etiopathogenesis, AI has recently made a significant contribution to our understanding of AD by identifying a new, unidentified etiologic factor that promotes skin inflammation in the paired related homeobox-1 (Prx1)+ fibroblastic subpopulation by overexpressing eotaxin-1: dysregulation of the inhibitor of nuclear factor kappa B kinase subunit beta-nuclear factor kappa-light-chain-enhancer of activated B cells (Ikkb-NF-kB) axis (Sivamaruthi et al. 2023).

ML approaches have been used to predict childhood AD onset using massive databases, including prenatal environmental contaminant exposure. The best predictive model was random forest (RF) (Huang et al. 2021). In 2023, new and efficient AD predictive models like bSRWPSO-FKNN were proposed by combining swarm intelligence algorithms (binary enhanced particle swarm optimization; bSRWPSO) with well-known supervised ML techniques (fuzzy K-nearest neighbor; FKNN) (Li et al. 2023). The automatic extraction of lesions and segmented skin images using DL models for accurate skin condition classification, including AD, provided an image dataset that improved the CAD performance of diverse skin disorders. (Yanagisawa et al. 2023). Interestingly, ML approaches (with the ideal model represented by Extreme Gradient Boosting; (XGB) are increasingly being used to accurately diagnose AD by utilizing biomarkers such pyroptosis-related genes (PRGs) (Wu et al. 2023).

Recent dermatologic imaging methods, such as 3D Raster-Scanning Optoacoustic Mesoscopy (RSOM), have shown encouraging results in testing AI applications for classifying AD and subclassifying its severity. These methods demonstrate high predictive accuracy in classifying AD for CNNs but are not as accurate for severity subclassification as the RF model (Park et al. 2021).

An increasing number of applications for AI are being developed to evaluate the relationship between external risk variables and the clinical deterioration of AD. Patella et al. found a proportionality between higher (SCORAD) scores and air pollution concentrations and total pollen counts in an observational study utilizing an ANN (Patella et al. 2020).

In the therapeutic realm, recent advances in DL-based models that use disease-specific gene expression profiles to generate new drug candidate molecules contributed to precision medicine (McMullen et al. 2023). AI, through platforms like Chat Generative Pre-Trained Transformer (ChatGPT) and specific mobile health apps, has also begun to play a key role in providing patients with clinically precise and comprehensive data on this disease, with psychopathological repercussions, particularly for parents with AD children (Lakdawala et al. 2023).

## Conclusions and prospects

Our understanding of AD’s pathogenesis’s, management, and epidemiology is improving, preparing us to fight this challenging disease. During certain windows of opportunity, the use of immunomodulatory drugs, emollients, education, and lifestyle modification can prevent AD and its associated comorbidities. Despite advances, the identification of serum or plasma biomarkers and the generation of further specialized treatments remain unfinished. Discovering these biomarkers could revolutionize AD pathogenesis and improve early diagnosis, prevention, treatment, and patient outcomes. The challenges in treating AD in children include its high prevalence, its unpredictable nature, and the limited number of effective medications with acceptable benefit-risk ratios. Continuous adjustments to treatment plans are essential for optimal disease management and symptom relief.

With the approval of dupilumab, moderate-to-severe atopic dermatitis can be treated more safely without the need for a wide range of systemic immunosuppressants. This has encouraged the development of new biologics and small compounds targeting the interleukin (IL)-4/IL-13 pathway, such as JAK inhibitors. Nemolizumab, a new anti-IL-31 receptor, may be used as a second-line treatment for pruritus after biologics fail. American FDA-approved drugs include upadacitinib and abrocitinib (JAKI). These therapies provide new alternatives for patients with AD.

JAKi is not a ‘one-size-fits-all’ AD treatment. The advantages, challenges, and ideal solutions for in vivo selectivity are unclear. The next-generation Jaki exhibits promising in vitro selectivity, but clinical trials are needed to confirm their selectivity. The FDA requires JAK inhibitors to contain “black-boxed warnings,” which list increased risks of deaths, cardiovascular disease, cancer, thrombotic events, and serious infections. JAK inhibition in atopic dermatitis can cause significant adverse effects; thus, pharmacovigilance is needed.

Advancements in nanotechnology have led to substantial enhancements in patient care and quality of life for AD patients. Nanoparticles may be a viable medication delivery method to overcome skin permeability and solubility limitations (Abdel-Mageed et al. 2021, 2022a). Nanoparticle formulations for topical medication administration for AD include antibiotics, corticosteroids, and herbal, synthetic, and herbal-synthetic combinations (Damiani et al. 2019; Abdel-Mageed et al. 2022b). This area offers new ways to track the disease course, evaluate recommended treatments, and track disease evolution. Despite the increased interest in nanoparticulate formulations to improve topically administered medication absorption and bioavailability, The FDA and EMA have not established topical use guidelines. Current regulations focus on parenteral nanomedicine formulations. However, this framework can be used to determine and study nanoformulation quality aspects like microbiological, chemical, and physical qualities, while producing medications. Additionally, nanotechnology-based remedies can cause allergic reactions and worsen symptoms, which requires constant monitoring (Abdel-Mageed et al. 2024).

Over the past 3 years, AI has been investigated for AD diagnosis to identify chronic skin conditions swiftly, automatically, and without an operator enabling drug repositioning, personalized dermatology and improved patient care, particularly for patients with difficulty accessing specialized treatments. Although AI can enhance dermatological care, ethics, training needs, and validation must be addressed first for proper integration. Cultural limitations make dermatologists reluctant to challenge their clinical knowledge or lose autonomy in clinical decision-making, questioning the human–machine partnership’s profitability. A noteworthy constraint of these research studies is their heavy dependence on datasets consisting chiefly of Caucasian skin types. Potential biases may exist because various ethnic groups may exhibit skin diseases in distinct physical ways (Rezk et al. 2022). When diagnosing or evaluating skin problems in non-Caucasian populations, the models may not function as well or properly if there is no representation of a range of skin tones (Cho et al. 2023).).

Finally, with more new AD medications in development, precision medicine may optimize benefit-risk ratios, especially in youngsters and the elderly, where drug–drug interactions are an issue. Preventive Medicine, comprehending AD’s immunological and genetic complexity, and managing co-occurring disorders are still being explored. The main points of concern are:

I. Low-impact prevention techniques to prevent AD in all at risk populations, especially children
II. Pathogenesis and etiology improved knowledge to aid in prevention
III. Endotypes with Easy definitions that are applicable to all types of organizations
IV. Epidemiological studies to include underprivileged populations in clinical trials and other research projects involving patient diversity
V. The nanotechnology field demands deeper persuasive studies on the toxicological and immunological effect of dosage forms

## Data Availability

Data generated or analyzed during this study are included in this published article.
